# Proceedings of Medical Societies

**Published:** 1864-04

**Authors:** 


### PROCEEDINGS OF MEDICAL SOCIETIES.

### MONTGOMERY COUNTY MEDICAL ASSOCIATION.

Pursuant to a call, the physicians of Montgomery Co., Ind., met in Crawfordsville, at the office of Dr. S. B. Morgan, on the 24th of October, 1863.

Dr. M. Herndon was called to the Chair, and Dr. E. W. Keegan appointed Secretary.

On motion of Dr. English, a committee consisting of Drs. Wilson, May and Detchon, was appointed to draft and present to the Society at its next meeting, a Constitution and By Laws.

On motion of Dr. Edgerlee, a committee consisting of Drs. E. Detchon, J. J. Shannon, J. G. McMechan, R. G. English, A. T. Steele, H. Keeney, and J. S. French, was appointed to prepare and present a schedule of prices for the government of the members of the Society.

Adjourned to meet Nov. 7, 1863.

Nov. 7th, 1863.

Montgomery County Medical Association met pursuant to notice at adjournment.

Minutes of the preceding meeting were read and approved

Reports from the committees on Finance, and on Constitution and By-Laws were read and approved.

On motion of Dr. English, the Association proceeded to the election of Officers for the ensuing year, resulting as follows:

President, Dr. M. Herndon; Vice Presidents, Drs. S. G. Irvin and R. G. English; Rec. Sec'y, Dr. E. W. Keegan; Cor. Sec'y, Dr. J. B. Wilson; Treasurer, Dr. E. Detchon.

Ordered, that the annual meeting of the Association, shall be held on the first Wednesday in January of each year, in Crawfordsville, and the quarterly meetings at such places as may be designated.

Committees were appointed to report at the annual meeting in 1865, on the following branches:

*Surgery*—G. W. Edgerlee, M. D., Chairman, S. G. Irwin, M. D., and J. B. Wilson, M. D.

*Theory and Practice*—R. G. English, M. D., Chairman, J. G. McMechon, M. D., and E. Detchon, M. D.

*Obstetrics*—S. B. Morgan, M. D., J. J. Shannon, M. D., and M. S. Bass, M. D.

*Materia Medica*—J. Sloan, M. D., J. McClelland, M. D., and A. F. Steele, M. D.

*Physiology*—Stowe Detchon, M. D., W. S. May, M. D., and E. W. Keegan, M. D.

*Chemistry and Medical Statistics*—J. W. Straughan, M. D., S. G. Irwin, M. D., and E. Detchon, M. D.

Ordered, that the proceedings be published in the *Chicago Medical Journal.*

Adjourned to meet January 7th, 1864.

E. W. KEEGAN, Rec. Sec.

---

### AURORA MEDICAL ASSOCIATION.

AURORA, Feb. 11th, 1864.

The Aurora Medical Association met at Dr. Young's office. The President and Vice President being absent, the Secretary, Dr. Young, called the Association to order, and on motion of Dr. Howell, Dr. P. A. Allair was chosen Chairman, *pro tem*

Dr. Howell proposed Dr. J. Woodworth for membersh ip who was duly elected, and signed the Constitution.

On motion of Dr. Higgins, the Association proceeded to the election of officers for the ensuing year, and the following named gentlemen were duly elected:

President, D. W. Young, M. D.; Vice President, P. A.

Allair, M. D.; Secretary, J. Woodworth, M. D.; Treasurer, A. D. Howell, M. D.

Dr. Young offered the following:

*Resolved*, That the undersigned, practitioners of Medicine of the city of Aurora, do hereby agree and pledge ourselves to each other, as honorable men, that we will not do professional business for any person or persons, who have failed or refused to pay or settle their medical bill to their Physician, and whose names are reported to us or either of us as having so failed or refused to pay such medical bills; and we each further pledge ourselves, one to the other, to report and place on our "BLACK LIST" the names of every person or persons so failing or refusing to Physicians signing this Resolution, and, that we will, under no circumstances, attend them nor their families, until they first furnish satisfactory evidence of their settling with their prior attending Physician.

On motion of Dr. Woodworth, it was unanimously adopted, and the Pres., Dr. Young, appointed to circulate this resolution and obtain the signatures of all the Physicians in the city.

On motion of Dr. Young, the Sec'y was instructed to furnish a copy of our proceedings to the *Chicago Medical Journal*, for publication.

On motion of Dr. Howell, the Association adjourned to meet at Dr. Young's office, Feb. 18th, 1864.

J. WOODWORTH, Sec.

---

ILLINOIS STATE MEDICAL SOCIETY.

The regular annual meeting of the Illinois State Medical Society will be held in Chicago, on the first Tuesday in May, 1864. Every regularly organized local Medical Society is entitled to send one delegate for every ten members, and an additional delegate for a fraction of more than half of ten. Each Medical College and Hospital is entitled to two delegates. We hope there will be a general attendance from all parts of the State.

N. S. DAVIS,

Permanent Secretary.

Chicago, March 5th, 1864.

---

AMERICAN MEDICAL ASSOCIATION.

The 15th Annual Meeting of the "American Medical Association" will be held in the City of New York, commencing on Tuesday, June 7th, 1864, at 10 o'clock A. M.

GUIDO FURMAN, M. D., Secretary.

New York, March, 1864.

